# Molecular and Epigenetic Aspects of Opioid Receptors in Drug Addiction and Pain Management in Sport

**DOI:** 10.3390/ijms24097831

**Published:** 2023-04-25

**Authors:** Filomena Mazzeo, Rosaria Meccariello, Ezia Guatteo

**Affiliations:** 1 Department of Economics, Law, Cybersecurity and Sports Sciences, University of Naples “Parthenope”, 80133 Naples, Italy; 2 Department of Movement Sciences and Wellbeing, University of Naples “Parthenope”, 80133 Naples, Italy; 3 IRCCS Santa Lucia Foundation, Via del Fosso di Fiorano 64, 00143 Rome, Italy

**Keywords:** opioid receptors, opioids, dopaminergic system, neuron excitability, drug addiction, nociception, epigenetics, ncRNAs

## Abstract

Opioids are substances derived from opium (natural opioids). In its raw state, opium is a gummy latex extracted from *Papaver somniferum*. The use of opioids and their negative health consequences among people who use drugs have been studied. Today, opioids are still the most commonly used and effective analgesic treatments for severe pain, but their use and abuse causes detrimental side effects for health, including addiction, thus impacting the user’s quality of life and causing overdose. The mesocorticolimbic dopaminergic circuitry represents the brain circuit mediating both natural rewards and the rewarding aspects of nearly all drugs of abuse, including opioids. Hence, understanding how opioids affect the function of dopaminergic circuitry may be useful for better knowledge of the process and to develop effective therapeutic strategies in addiction. The aim of this review was to summarize the main features of opioids and opioid receptors and focus on the molecular and upcoming epigenetic mechanisms leading to opioid addiction. Since synthetic opioids can be effective for pain management, their ability to induce addiction in athletes, with the risk of incurring doping, is also discussed.

## 1. Introduction

For a long time, opioids from *Papaver somniferum* have been used recreationally as a euphorigenic or medicinally as analgesic for the treatment of pain [1]. Natural opioids, but also several newly developed synthetic opioid drugs, interfere in the endogenous opioid system. This complex signaling system comprises endogenous peptide-ligands that bind to specific receptors to produce analgesia, reward and pleasure [2]. Opioid receptors include four classes of G_i/o_-protein coupled receptors, named mu-(MOR), kappa-(KOR), delta-(DOR) opioid receptors and the most recently described nociceptin opioid peptide (NOP) receptor [3]. The endogenous ligands of MOR and DOR are enkephalins and endorphins; KOR are activated by endogenous dynorphin peptides [4,5]; NOP receptors are activated by endogenous nociceptin opioid peptide. Dynorphins include several products (dynorphin A, dynorphin B, big dynorphin, etc.) originating from a pro-peptide pro-dynorphin that can be processed differently in the diverse brain areas [6,7]. It is currently unknown which dynorphin is released to activate KOR in the midbrain and other brain areas [8].

Today, opioids are still the most used and effective analgesic treatments for severe pain, but their use and abuse cause detrimental side effects for health, including addiction, thus impacting the user’s quality of life, increasing the risk of opioid use disorder (OUD) and causing overdose [9]. In this respect, drug addiction is a complex, chronic, relapsing brain disorder that centrally involves several neural networks in the development of drug addiction and relapse after withdrawal from drugs of abuse (e.g., the mesocorticolimbic system as reward system, the extended amygdala as anti-reward/stress system and the central immune system for neuro-inflammation) [10]. In general, occasional use, recreational use, regular use and addiction are the main developmental steps in drug addiction [10,11]. The neuronal population involved in addiction process comprised dopaminergic neurons that project from the ventral tegmental area (VTA) to the Nucleus accumbens (NAcc), cortical region and amygdala, hippocampus and pre-frontal cortex. Opioid signaling in dopaminergic neurons is critical for the development of drug addiction. In fact, in the VTA, the opioid-dependent activation of MORs elevates dopamine signaling in the NAcc; direct effects via MORs have been also reported in both forebrain and NAcc; lastly, the activation of KOR or DOR in distinct neuronal and non-neuronal cells interferes in different signaling systems, causing long-lasting adaptions to cellular functions that promotes behavioral and psychological abnormalities underlying addiction [12].

Hence, the knowledge of opioid system modulation and signaling may be useful to better understand changes in brain circuits functioning and for the development of suitable therapeutic strategies in the treatment of brain diseases. In this respect, epigenetics is a relatively new area in genetics focused on the modulation of gene expression by lifestyle and environmental factors, without any change in DNA nucleotide sequence [13]. Epigenetic mechanisms include different strategies that aim to modulate the chromatin architecture and the subsequent DNA accessibility to transcription. The main epigenetic mechanisms are: (1) DNA methylation at cytosine (C) residues located within specific GpC sites in promoter regions; (2) chemical modifications of histone (H) tails that remodel euchromatin vs. heterochromatin architecture and vice versa; (3) the production on non-coding RNAs (ncRNAs) (e.g., microRNA (miRNA), long non-coding RNA (lncRNA), tRNA fragments (TRFs), circular RNA (circRNA), etc.) [14,15,16,17,18]. Stable epigenetic modifications in specific neuronal populations occur because of persistent adaptation and are proportional to the nature and long-lasting of the experience of addiction [12,19,20]; similarly, changes in the epigenetic signature occurs in nociception leading to analgesic tolerance and dependence [21,22,23]. Hence, in recent years, epigenetic signature has been investigated centrally and at the periphery, providing evidence of new molecular pathways and new diagnostic, prognostic and therapeutic perspectives for the treatment of addiction. 

In this review, we summarize the main features of opioids, opioid receptors and excitability changes induced by their activation in midbrain dopaminergic neurons and focus on the molecular and upcoming epigenetic mechanisms leading to opioid addiction. Since synthetic opioids can be effective for pain management, their ability to induce addiction in athletes, with the risk of incurring doping, is also discussed. 

## 2. Opioids and Opioids Receptors

Over 500,000 deaths have been ascribed to opioids since the mid-1990s, with some attributed to unstable pain management [1]. The addiction to illicit opioids and the misuse of prescription synthetic opioids pain relievers and fentanyl analogs generated an opioid epidemic in North America over the past two decades. This then moved to Europe, with a significant increase starting from 2015 involving mainly northern and eastern countries, also in addition to the Mediterranean area [24]. 

Opioids are a large class of substances related in structure to the natural plant alkaloids found in opium that are derived from the resin of the opium poppy, *Papaver somniferum*, an herbaceous plant native to Turkey but now present in all temperate climate areas [25]. The natural alkaloids are also referred to as opiates and include morphine and codeine. Synthetic derivatives include heroin, fentanyl, hydromorphone, methadone, buprenorphine and others. 

Several opioids are available for clinical use, including morphine, hydromorphone, levorphanol, oxymorphone, methadone, meperidine, oxycodone and fentanyl, and their advantages and disadvantages for the management of pain have been discussed [26]. Opioids have been the mainstay of pain treatment for thousands of years, and they remain so today. Moreover, opiates derived from opium include the natural products morphine, codeine and thebaine, and many semisynthetic derivatives. 

Opioids such as heroin (a substance of abuse) and morphine exert their effects by mimicking naturally occurring substances called endogenous opioid peptides or endorphins [25]. Endogenous opioid peptides are the naturally occurring ligands for opioid receptors [25]. The term endorphin is used synonymously with endogenous opioid peptides but also refers to a specific endogenous opioid, β-endorphin. The term narcotic was derived from the Greek word for “stupor”. At one time, the term referred to any drug that induced sleep, but then it became associated with opioids. It is often used in a legal context to refer to a variety of substances with abuse or addictive potential [27].

In the endorphin superfamily, three major systems of endorphin peptides (pro-opiomelanocortin, proenkephalin and prodynorphin) and no less than two populations of minor opioid peptides (the endomorphins and the orphanin/nociceptin peptide) exist in independent neuronal circuits [25]. On the basis of structural homologous with other family members, the opioid receptor-like protein (NOP) is cloned; it is coupled to a G protein and has an endogenous ligand, nociceptin/orphanin FQ (N/OFQ), but it does not exhibit the pharmacological profile of opioids [28].

Opioid substances comprise all natural and synthetic alkaloid derivatives of opium. These natural opioid peptides arise from independent but homologous genes. The peptides all share some actions at the receptors, but these are now undergoing progressive refinement.

The human opioid system contains four opioid receptors (MOR, DOR, KOR and NOPR (nociceptin receptor)) and a set of related endogenous opioid peptides (EOPs), which show distinct selectivity toward their respective opioid receptors (ORs).

Opioid receptors appear early in the evolutionary process of vertebrates [29]. MOR, DOR and KOR share extensive sequence homology (55–80%). They belong to the rhodopsin family of G protein-coupled receptor (GPCR) and represent the largest group of membrane proteins encoded in the human genome. The opioid receptors in humans have been mapped to the 1p355-33 chromosome (DOR) on the 8q11,23-21 chromosome (KOR) on the 6q25-26 chromosome (MOR) [30].

All opioid receptors are widely distributed in the central and peripheral nervous system. They are found in the brain, the spinal cord, some peripheral sensory neurons and the gastrointestinal tract [31]. They are activated by EOPs to execute various physiological effects including intrinsic analgesia.

Ligands for opioid receptors can be generally defined on the basis of their functional properties as agonists and antagonists, as summarized in Table 1 and Table 2.

Additional receptor types were proposed (e.g., sigma, epsilon, orphanin) but they are no longer considered “classical” opioid receptors [32,33].

Opioids have been used in pain management since ancient times and are the most potent drugs for the treatment of severe pain. Opium is one of the oldest medicines in use as an analgesic and sedative [34].

Consistently, with the expression of opioid receptors at all levels of the neuraxis, opioid agonists can efficiently inhibit clinical pain after peripheral (topical, intraarticular), neuraxial (intrathecal, epidural, intracerebroventricular) or systemic (intravenous, oral, subcutaneous, sublingual, transdermal) administration [35]. The commonly available opioid drugs (morphine, codeine, methadone, fentanyl and their derivatives) are primarily MOR agonists. The choice of a particular compound or formulation is based on pharmacokinetic considerations (route of administration, *compliance*, distribution) and on side effects. The administration of spinal opioids with systemic opioids to manage pain can produce similar side effects (e.g., excessive sedation, respiratory depression, nausea, vomiting, constipation, pruritus, and other adverse effects), depending on dosage and rostral/systemic redistribution [36,37].

Opioids, depending on their receptor preferences, produce a series of effects consistent with the role of the apparatuses to which the receptors are associated [30]. Although the primary clinical commitment of opioids is related to their pain-relieving properties, they produce a number of other effects. This is not surprising considering the wide distribution of opioid receptors in the brain, spinal cord and peripheral systems.

Regarding the nervous system, the actions of opioids range from analgesia to effects on motivation and complex behaviors, such as euphoria, alertness and numerous autonomic hormonal and motor processes. In peripheral systems, opioids may affect gastrointestinal tract motility and smooth muscle tone (Table 3).

## 3. Neuronal Networks and Mechanisms in Opioid Addiction

### 3.1. The Dopaminergic System

**Figure 1:**
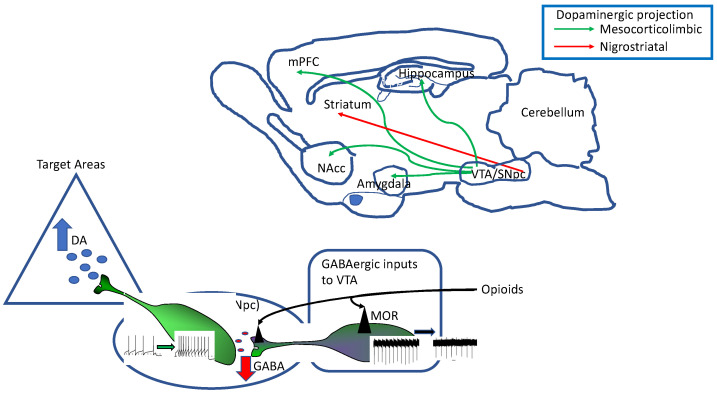
(**Top**) Schematic representation of a parasagittal rat brain section with depicted the main dopaminergic pathways, originating from the ventral midbrain nuclei, the ventral tegmental area (VTA) and the substantia nigra pars compacta (SNpc). The mesocorticolimbic pathway (green arrows) originate mainly from the VTA and represents the major reward system in the brain. (**Bottom**) Functional connections between VTA and its target areas are strongly affected by inhibitory (GABAergic) inputs to VTA originating from different brain areas. The most robust effect of opioids is linked to inhibition of GABAergic neuron firing and consequent disinhibition of VTA DA neurons that increase firing rate and DA release.

The mammalian midbrain hosts two adjacent nuclei containing the vast majority of the dopamine-synthetizing/-releasing neurons in the brain, the substantia nigra pars compacta (SNpc) and the ventral tegmental area (VTA; Figure 1). Functionally, the SNpc is part of the basal ganglia, a group of five interconnected nuclei controlling voluntary movement which comprehend the striatum (caudate and putamen), globus pallidus (external and internal), substantia nigra (compacta and reticulata) and subthalamic nucleus. Recently, other two nuclei have been included in the basal ganglia: the peduncolopontine nucleus and the zona incerta [61,62]. The VTA is part of the mesocorticolimbic system, a major motivation and reward system in the brain consisting of dopaminergic projections from VTA to cortical and subcortical target areas and it represents the brain circuit mediating both natural rewards and the rewarding aspects of nearly all drugs of abuse [63,64,65]. 

The two dopaminergic (DAergic) systems participate in general brain functions, such as movement, learning and memory, motivation, emotional behavior and cognition, by regulating the amount of dopamine (DA) released throughout the forebrain [66,67,68,69,70].

As a consequence, dysfunctions of the DAergic system or modifications of DA neurons excitability lead to alterations to DA release in the different brain areas, causing many neurological and psychiatric disorders, such as Parkinson’s disease, schizophrenia, attention-deficit hyperactivity disorder and drug abuse. Within the context of the present review, midbrain DA neurons and their projections to diverse brain areas are responsible for mediating the reward of nearly all drugs of abuse, including opioids [71].

In neurons, neurotransmitter release by pre-synaptic terminals strongly depends on the shape, frequency and pattern of somatic action potentials, generated by intrinsic neuronal membrane ion fluxes and/or in response to synaptic inputs [72,73]. Particularly, the action potential duration dramatically affects the amount of neurotransmitter release by regulating the entity of calcium influx into pre-synaptic terminals, which in turn controls the amount of neurotransmitter vesicle exocytosis within the synaptic cleft [73].

Different neuronal populations display peculiar electrophysiological properties that confer specificity to that neuron type(s), and the modification of these properties can enormously affect the relevant physiological functions of that particular nucleus [74]. 

DA neurons located in the SNpc and VTA are characterized by spontaneous action potential firing, either as the tonic regular pacemaker (single spike) or as phasic burst firing (as recently reviewed by [75]). The latter has been mainly described in vivo, as it depends on glutamatergic circuitry impinging onto DA neurons, whereas primary cultured DA neurons or those maintained in ex vivo brain slice preparations mainly fire as regular pacemakers [76,77]. Bursting activity, consisting of clusters of two-to-ten action potentials, causes DA release either at the distal sites [78,79,80] or locally, within the midbrain [81,82,83]. DA neurons express metabotropic D_2_-like autoreceptors [84,85] both in the somatodendritic compartment or axon terminals, whose activation by DA gates G protein-dependent inward rectifier potassium channels (GIRK) and the transient interruption of spontaneous firing [83,86,87]. The extracellular level of DA and the degree of D_2_-like autoreceptor activation is also regulated by its uptake via DA transporter (DAT), promptly activated by extracellular DA, representing the limiting factor in the control of peak and duration of the DA-GIRK current [83,88]. Alterations of DA neuron firing due to (i) changes of intrinsic membrane currents, (ii) an unbalance between excitatory/inhibitory neurotransmission or (iii) DAT activity would result in modifications of DA release at distal sites.

### 3.2. Opioid Receptors Expression by Dopaminergic Neurons 

NOP receptors are widely expressed in the mesencephalic area of rodents [89]. Particularly, about 50% of DA neurons of the VTA and SNpc, identified by the expression of DA synthesis rate-limiting enzyme tyrosine hydroxylase (TH^+^), express NOP mRNA and about 80% of NOP + cells are dopaminergic (TH^+^) [90]. The current hypothesis is that, within the midbrain, VTA/SNpc DA neurons express post-synaptic NOP receptors that are activated by NOP released by local GABAergic interneurons [91]. 

Although SNpc and VTA lack dynorphin-expressing neurons, significant peptide levels are found in these areas [92,93,94]. Accordingly, dynorphin-releasing fibers within the VTA originate from the dorsal and ventral striatum, amygdala and lateral hypothalamus [95]. In contrast, KOR binding sites and KOR mRNA is present in the VTA and SNpc [96,97], suggesting the presence of KOR-expressing neurons in these areas. Particularly, by means of retrograde labelling, it has been shown that VTA DA neurons projecting to NAcc mainly express KOR-mediated responses, whereas those projecting to BLA mainly express MOR/DOR-mediated responses [63].

In humans and animal models, DORs are expressed in cortical areas, hippocampus, amygdala, hypothalamus and basal ganglia [98]. Two isoforms of functional DOR have been described (DOR1 and DOR2) and, with regard to the midbrain, the vast majority of VTA DA (and non-DA) neurons express combinations of DOR1, DOR2 and/or MOR [99]. Indeed, evidence indicates that DOR and MOR interact in VTA DA neurons to alter downstream signaling. Additionally, direct measure of DOR expression in VTA DA neurons by means of different techniques [99] showed that DOR1 mRNA is present either in DAergic or non-DAergic VTA neurons. Interestingly, most of the neurons tested also expressed MOR mRNA, suggesting the co-expression of DOR and MOR in many VTA DA neurons. 

MOR activation mediates either analgesic or rewarding effects and are expressed by VTA DA neurons, as well as by astrocytes [100]. MOR are also expressed in GABAergic terminals from rostromedial tegmental nucleus (also named tail of the VTA), impinging onto VTA and substantia nigra DA neurons where they mediate the inhibition of transmitter release [101,102]. MOR are also expressed by most of VTA TH^+^ neurons, as revealed by the co-expression of MOR and TH mRNAs in most of the neurons tested [103]. 

### 3.3. Mechanisms of Opioid Receptor Modulation of DA Neuron Function

#### 3.3.1. Enkephalinergic Modulation of DA Neuron Intrinsic Excitability

The reinforcing properties mediated by opioids are linked to their complex actions on the VTA circuitry. Due to high heterogenicity of this neurocircuit, the overall responses of DA neurons to opioids are diverse [103]. As revealed by rodent studies on cellular and circuitry functions, opioids indirectly stimulate DA release (and DA neuron firing) by silencing GABAergic interneurons that exert a tonic inhibition onto DA neurons (Figure 1) [104,105]. Opioids also regulate inhibitory inputs to VTA from different brain areas [105] and glutamate release onto VTA neurons [106,107]. However, other than modulating synaptic circuitry impinging onto DA neurons, opioids also modulate directly intrinsic excitability of these cells. 

KOR are critical players in modulating DA neurons activity and release of DA [63]. Indeed, activation of KOR located at somatodendritic compartment gates a K^+^ conductance that hyperpolarize both VTA and SNpc DA neurons [108,109,110], although inhibition is stronger in VTA than SNpc neurons. Additionally, KOR directly inhibit DA vesicle release [111], suggesting that KOR acts at both pre- and postsynaptic sites.

Within the VTA, the effects of KOR activation on DA neurons’ excitability is different depending on the specific VTA neuronal populations projecting to diverse target areas (Table 4).

Particularly, VTA neurons projecting to NAcc are not hyperpolarized by KOR agonists [110], whereas NAcc medial-shell and lateral-shell projecting neurons are inhibited by KOR [63,108], as well as mPFC- and BLA-projecting VTA neurons [115]. The KOR agonist U-69593 activated a GIRK-mediated outward current in TH+ and Ih-expressing DA neurons, whereas it was ineffective in TH^−^ neurons [109]. It has been proposed that the outward (inhibitory) current elicited by the same KOR agonist in mPFC-projecting VTA DA neurons may underlie the aversive effects mediated by KOR agonist when directly applied to the VTA in mice [118]. Recently, it has been proposed that GIRK activation by KOR may not simply serve as firing inhibition, but it may also promote integration of excitatory inputs [8].

The most robust effect of opioids acting at MOR measured in DA neurons is pre-synaptic inhibition of GABA release which results in disinhibition and firing increase of these neurons (Figure 1) [104]. At least two types of GABA-ergic neurons are affected by opioids, local interneurons within the VTA, directly hyperpolarized by MOR, responsible for GABA-A mediated post-synaptic currents onto DA neurons, and GABAergic fibers originating in the ventral pallidum or NAcc, that mediate GABA-B post-synaptic currents, affected by both MOR and KOR agonists (reviewed by [71]). Additionally, MOR have been shown to decrease the inhibitory tone onto VTA DA neurons exerted by tail-VTA (tVTA) [113]. GABAergic neurons causing DA neurons disinhibition/excitation, increase DA release within the NAcc shell, and this has been proposed to be a plausible reward mechanism of drugs of abuse, acting at MOR like heroin [101,104,119]. 

MOR decreased both inhibitory and excitatory inputs to selective VTA DA neurons: those projecting to the NAcc medial shell, causing an indirect increase of action potential firing in these cells [114]. Within the midbrain, secondary (GABAergic) and tertiary (DA-, 5-HT- and Met-enkephalin-sensitive) cells were described to be directly hyperpolarized by MOR activation [71,112]. More recently, it has been shown that in VTA neurons, MOR can form heterodimers with DOR1 and DOR2, thus differentially coupling to downstream signaling pathways. Thus, either the MOR or DOR1/2 agonists produced either a predominant K^+^ dependent hyperpolarization or a Cav2.1 mediated depolarization in different VTA neurons [99].

The DOR1 agonist elicited different responses in DA neurons of the VTA, causing hyperpolarization, depolarization or no change of membrane potential [99]. Similarly, the activation of DOR2 caused either hyperpolarization or depolarization of membrane potential in VTA DA neurons. The mechanisms underlying the cellular responses involve the activation of K^+^ and Ca^2+^ conductances, respectively, thus posing for excitatory (not only inhibitory) neuronal responses mediated by opioid receptors. This evidence suggests that the vast majority of VTA DA neurons do express post-synaptic DOR1, DOR2 isoforms and MOR. The heterogeneity of cellular responses may depend on the agonist activation of homo- vs. hetero-dimers, as interaction between DOR and MOR have been postulated. DOR located on pre-synaptic GABAergic terminals in the VTA have been shown to inhibit GABA release selectively in low ethanol-drinking rats and this was correlated with behavioral inhibition of ethanol consumption [115]. 

The contribution of DOR in reinforcing mechanisms of drugs of abuse is linked to their modulation of DA release in the VTA and NAcc [2]. DOR contribute to reinforcing mechanisms of different drugs of abuse, including morphine, cocaine and nicotine [2,120].

With regard to the cellular actions mediated by NOP activation by nociceptin/orphanin N/OFQ, they resemble those mediated by the other classes of opioid receptors, particularly the activation of K^+^ conductances, the inhibition of high threshold voltage-gated Ca^2+^ channels and the inhibition of transmitter release [121]. While most of VTA neurons responded with large outward currents to NOP activation [116], a recent study found that, NOP effects on the intrinsic membrane currents have different features depending on the projection areas of VTA and SNpc DA neuronal populations [117]. Medial-PFC and NAcc-projecting VTA neurons respond to NOP activation with an outward (inhibitory) membrane current; posterior anterior cingulate cortex (pACC)-projecting VTA neurons responded to NOP activation with an inward (excitatory) membrane current [117]. The outward current is not K^+^-dependent but GABA-A mediated. Thus, NOP act in VTA neurons by increasing GABA-A mediated currents.

#### 3.3.2. Epigenetic Mechanisms and Targets in Drug Addiction

Stable epigenetic modifications in specific neuronal populations occur because of persistent adaptation and are proportional to the nature and long-lasting of the experience of addiction [12,19,20]. 

**Figure 2:**
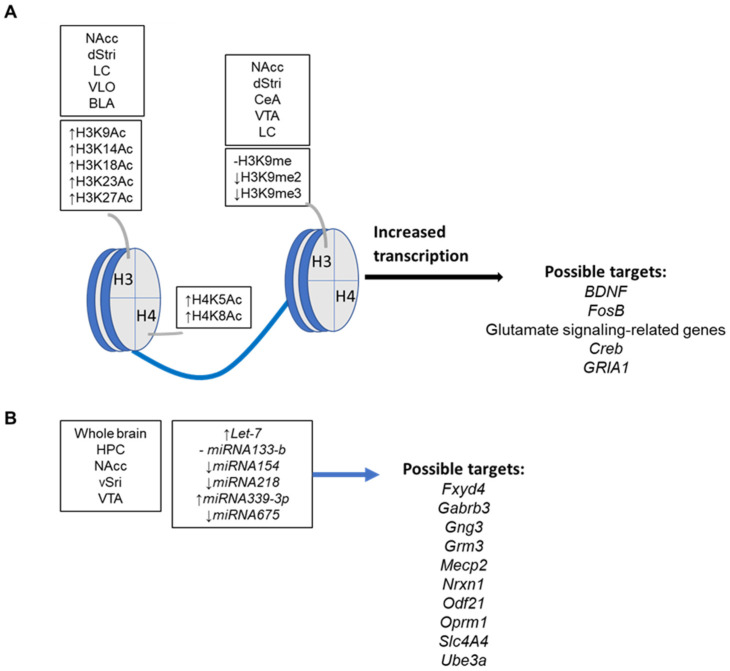
(**A**) Main histone tail changes caused by long-term opioid exposure on specific brain areas in rodents and humans. (**B**) Main deregulated miRNA and targets in specific brain areas following long-term opioid exposure in mouse models. Basolateral and central amygdala (BLA and CeA, respectively), dorsal stiatum (dStri), hippocampus (HPC), locus coeruleus (LC), nucleus accumbens (NAcc), ventral stiatum (dStri), ventral tegmental area (VTA), ventrolateral orbitofrontal cortex (VLO). Data extracted from [12]. -, stable levels; ↑, increased levels; ↓, decreased levels.

The main opioid dependent epigenetic changes are the acetylation of specific Lys (K) residues in the tail of H3 and H4 (i.e., H3K9Ac, H3K14Ac, H3K18Ac, H3K23Ac, H3K27Ac, H4K5Ac, H4K8Ac), histone di- and tri-methylation at H3K9 (i.e., H3K9me2, H3K9me3) and H3K27 (i.e., H3K27me3) gene- specific changes in DNA methylation status at CpG sites and the production of non-coding RNAs [12]. To date, KOR receptor gene (*OPRK1*) was the first found epigenetically modulated at chromatin levels by stress and nerve growth factor (NGF) [122]; in P19 mouse embryonal carcinoma cells, mechanisms involving chromatin changes in promoter region (i.e., demethylation at Lys9 and dimethylation at Lys4 of histone H3), transcription factor activation protein 2beta (AP2b) and PI3K system were reported [122]; in hippocampal cell lines Ht22, involvement of transcription factor Myc has been also demonstrated [123]. However, the activity of epigenetic writers or erasers like the histone acetyltransferases (e.g., HATs), histone methyltransferases (e.g., HMTs like G9a), histone deacethylases (e.g., HDACs), histone demethylases (e.g., HDMs) de novo, ex novo or maintaining DNA methyltransferases (e.g., DNMTs), DNA oxydases or DNA demethylases (e.g., ten eleven translocation proteins, TETs) has been reported in specific brain areas and recently reviewed [12]. Similarly, several transcriptome-wide changes in gene expression following long-term opioid exposure and the changes in the epigenetic marks were reviewed recently [12]. A summary of the main opioid exposure related changes in the epigenetic landscape of neurons is in Figure 2.

Recently, the role of ncRNA in the modulation of gene expression was demonstrated. NcRNAs comprise a large set of RNA not used for protein translation, and include miRNA, lncRNA, piRNA, TRF or circRNA [14,15,16,17,18]. They exhibit a tissue specific expression rate, but they are also encapsuled in specific vehicles such as microvesicles or exosomes to be released in tissues and biological fluids such as blood [124]; in such a way, ncRNA may modulate gene expression in cell types far away the site of origin and represent a biomarker in several pathological conditions, including opioid addiction [125]. 

The most studied ncRNA are surely miRNA, 22nt long single-stranded RNA that are capable to bind miRNA response elements in the 3′untranslated region of target mRNAs, thus repressing their translation or causing their degradation [126,127]. 

Apart from miRNAs, in recent years, particular attention has been reserved for lncRNA and circRNA. LncRNAs are a heterogeneous class of more than 200 nt in length ncRNAs classified as intronic, intergenic, bidirectional, antisense and sense, due to the localization of the corresponding genes in introns, intergenic regions or partially overlapping exons localized both on the forward and reverse strands [128]. They modulate gene expression at the transcriptional and post transcriptional level and establish a network of interactions with miRNA pathways [129]. CircRNAs are the result of back splicing of exons from pre-mRNAs, lack free 5′ or 3′ ends and sometimes maintain the ability to encode proteins. They affect gene expression at the transcriptional or post-transcriptional level, regulate the splicing process, the translation and stability of mature mRNAs in the cytoplasm, interfere with signaling pathways, act as molecular sponges for miRNAs, and may serve as templates for translation [130,131]. In the brain, lncRNA and circRNA resulted de-regulated in several neuroglia and astrocyte mediated neurological diseases, such as neuropathic pain, stroke, epilepsy, traumatic brain injury, spinal cord injury, ischemia-reperfusion injury, Alzheimer’s disease, multiple sclerosis and Parkinson’s disease [132].

The expression profile of ncRNA, particularly miRNA, has been largely investigated within brain area in both physiological and pathological conditions including drug addiction, as recently reviewed [12,19,20]. Hence, in this manuscript, we focus on upcoming data from the ncRNA world only.

Morphine is the main opiate analgesic drug used to relieve both acute and chronic pain [133] but is also a recreational drug due to its widespread availability and high addictive potential [134]. The molecular mechanisms underlying pathogenesis of morphine addiction are not fully understood, but experimental data from cell lines and animal models revealed that mitochondrial dysfunction occurred in the morphine addiction process, thus causing autophagy of DAergic neurons [135]. Signaling pathways involving the *lncRNA maternally expressed gene 3* (*MEG3*), Orexin1 receptor (OX1R), *c-fos* proto-oncogene, ERK and PKC were reported in mouse hippocampal neuronal HT-22 cells [135]. 

As reported in mirBase, in mammals, more than 700 miRNAs are expressed in the brain [136], but their functional role is not fully understood. MicroRNA and DOR receptor are well-known modulators of neuroinflammation and potential targets for the development of neuroprotective strategies to preserve neuronal functions [137,138]. Addictive drugs regulate the proliferation, differentiation and survival rates of adult neural stem/progenitor cells (NSPCs) with several interrelated pathways [139]. In such a context, an intriguing question is the involvement of opioid signaling in adult neurogenesis through the activation of miRNA-related pathways. KOR receptor agonists inhibit adult neurogenesis in mouse hippocampus up-regulating the expression rate of *miRNA7a*, which in turn modulates the activities of Pax6/Neurog2/NeuroD1 [140]. Consistently, the specific MOR receptor antagonist, Cys(2)-Tyr(3)-Orn(5)-Pen(7)-amide locked the differentiation of hippocampal neural progenitor cell lines (hNPCs) into astrocytes occurring via *miRNA181a*/Prox1/Notch1 following in vitro and in vivo morphine treatment [141]. 

A combined in vivo/in vitro study by Jan and coworkers [142] revealed that morphine dependence increased the expression of *miRNA132*—a CREB-induced and activation-dependent microRNA—in the dentate gyrus of rats promoting dendritic branching and spinogenesis; in turn, the inhibition of *miRNA132-3p* but not *miRNA132-5p* relieved morphine withdrawal symptoms. Consistently, in an in vitro model of μ-N2a cells that stably express MOR receptor, 24 h morphine treatment up-regulated *miRNA132*, promoting their differentiation [142]. Thus, *miRNA132* in the hippocampus participates in morphine dependence modifying neuronal plasticity. Conversely, morphine induces apoptosis of hippocampal neurons HT-22 with mechanisms involving the upregulation of *miR-181-5p* and the suppression of MAPK1 [143]. 

MiRNAs participate in the molecular pathways that modulate the behavioral phenotypes and the cellular neuroadaptations induced by opioid exposure. A recent study conducted in a rat model of morphine self-administration identified several drug-responsive microRNAs in the NAcc and revealed that their lasting expression profiles correlate with morphine-taking but not morphine-seeking behavior in males, since the miRNA profile undergoes regulation from early to late abstinence [144]. Therefore, miRNA signature may represent a marker of past drug abuse. 

Following microarray screening in the NAcc to find miRNAs responding to chronic heroin administration, *miRNA218* resulted the main actor in the inhibition of heroin-induced behavioral plasticity; at the molecular level, the direct inhibition of methyl CpG binding protein 2 (Mecp2) was also observed, and findings were confirmed in Mecp2^308/y^ mice [145]. This mouse model of Rett syndrome expresses a truncated form of methyl-CpG-binding protein 2 (*Mecp2*) gene [146]. An additional mechanism in heroin craving requires the interplay between MeCP2 and *miRNA181a*, the miRNA capable of directly binding the 3’UTR region of *Mecp2* [147]. Similarly, the overexpression of *miRNA9* in the NAcc significantly increased the self-administration of the synthetic opioid oxycodone (OxyContin in the market), increased “burst” episodes of intake and decreased the inter-infusion interval; this molecular pathway involves the decreased expression of RE1-silencing transcription factor (REST) and the increased expression of DA D2 receptor (DRD2) [148].

In recent years, the knowledge on cell-to-cell communications in the brain enlarged with the discovery of extracellular vesicle such as exosomes and microvesicles, membrane-bound vesicles capable of circulating in blood that delivers several cargos, ncRNA included [124]. Using RNA-Sequence analysis, distinct miRNA signatures and the related targets (e.g., *miRNA384-5p*, *miRNA30c-1*, *miRNA 195-5p*, *miRNA6318*, *miRNA666*, *miRNA128-1-5p*, *miRNA143-5p*, *miRNA3552*, *miRNA490-3p*, *miRNA1983*, *miRNA-3p* and *miRNA504*) were found deregulated in brain-derived extracellular vesicles after pre-natal and post-natal exposure to oxycodone. Key functional pathways associated with synaptic functions and affected by opioid exposure during prenatal and post-natal neurodevelopment and the possibility of disease load in later life were reported [149]. Exosomal miRNA and neurotransmitter profiles during heroin and methamphetamine withdrawal were investigated in humans in association with psychiatric comorbidities in patients with substance use disorders. This cross-sectional study enrolled 60 heroin-dependent patients, 60 methamphetamine-dependent patients and 20 controls from a joint program for drug detoxification and rehabilitation [150]. This study made it possible to identify in plasma a series of conserved and novel exosome-associated miRNAs with developmental and intellectual abnormalities that represent a molecular “fingerprint” of the progression of substance withdrawal. 

Additionally, circRNAs are gathering growing interest in the control of gene expression in specific brain regions. Arrays were recently used to identify heroin-responsive circRNAs in the rat orbitofrontal cortex, the brain region that mediates behavioral responses to rewarding stimuli. A total of 76 circRNAs resulted differently expressed vs. control rats and the deregulated and validated marks included *circAnks1a*, *circSlc24a2*, *circGrin2b*, *circAdcy5* and *circUbe2cbp*, corresponding to the linear mRNA encoding for ankyrin repeat and sterile alpha motif domain containing 1B, solute carrier family 24 member 2, glutamate ionotropic receptor NMDA type subunit 2B, adenylate cyclase 5 and ubiquitin protein ligase E3D, notably involved in cytoskeleton remodeling, transmembrane transport, cell signaling and cellular protein recycling [151]. *CircAdcy5* and *circGrin2b*, but not *circSlc24a2*, *circAnks1a* and *circUbe2cbp*, were co-regulated with their linear mRNA. Target miRNAs downstream of heroin-associated circRNAs were also identified using an integrated bioinformatics approach, thus revealing different mechanisms in the neurobiological adaptations that arise from chronic heroin exposure [151].

In an experimental model of morphine-conditioned place preference, the circular RNA of *tomoregulin-1* (*Tmeff-1*) gene (mmu_circ_0011663, *circTmeff-1*), resulted in being positively associated with the incubation of context-induced morphine craving, acting as a molecular sponge for *miRNA541-5p* and *miRNA6934-3p* in mouse NAcc core [152]. In parallel, the expression of *miRNA592-3p* within the NAcc resulted in being critical for the expression of just TMEFF1, since the overexpression of *miRNA592-3p* in the NAcc core decreased the expression of TMEFF1, consequently reducing the incubation of morphine craving [153]. Thus, a double route involving the *miRNA592-3p-*regulated expression of TMEFF1 and the *circTmeff-1* dependent modulation of *miRNA541-5p* and *miRNA6934-3p* may represent a critical step in opioid craving. Considering that morphine craving is the main sign of drug addiction that significantly contributes to a persistent vulnerability to relapse, the targeting of *circTmeff-*1 or *miRNA592-3p* may represent the input for possible therapeutic strategies in prolonged and forced abstinence from opioids.

Persistent and unwanted memories are thought to be critical contributors to drug addiction and the problem of chronic relapse over the life of people who are addicted. For a long time, memories have been considered static and fixed, but recently, new studies demonstrated that memories were dynamic and changeable under specific conditions [154]. In this respect, *circTmeff-1* activity in the NAcc was investigated in the reconsolidation of cocaine-associated memory, revealing a new role in decoy for *miRNA206* to regulate the expression of Brain-Derived Neurotrophic Factor [155]. Table 5 summarizes the main emerging de-regulated ncRNA in the complex process of drug addiction.

## 4. Opioids and Pain Management in Sport

Pain is present in almost all clinical pathologies and its management is a primary clinical imperative [156]. Opioids are more effective in reducing acute pain than chronic pain. Prescription opioids generally are mostly used to treat moderate to severe pain, but they are used similarly as medicines because they contain chemicals that relax the body. Nature and industry have produced a class of opioid drugs that alleviate pain and induce feelings of well-being [157,158].

The use of pain killers is frequent in sports, especially among athletes engaged in violent activities, such as boxing and combat sports in general [159]. Over and over again, the fear of losing a place leads the athlete to an addiction to maintain the fight in spite of any type of wound or handicap. The opium poppy alkaloids and their synthetic derivatives interact with receptors in the central nervous system, which are normally stimulated by endogenous neurotransmitters of the endorphin system [26]. These have the ability to moderate pain and also to control emotions. One must be aware of physical and psychological addiction induced by many opiates which are reasonably classified as narcotics. Narcotics have been on the “prohibited list” from the World Anti-Doping Agency since the list was created in 1967 [160,161]. Using drugs to improve performance (doping) in sport may lead to athletes being banned and damage to their health. A state resulting from periodic and repeated engagement of narcotics induces the subject to feel the need to continue drug intake and to increase the dosage to reach the same state or more pronounced effects [9,54,162].

An interruption in consumption leads to a withdrawal syndrome, which is more or less severe, depending on the substance. Frequent use can induce tolerance and dependence of variable severity depending on the type. Unfortunately, overprescribing and misuse of these drugs pose serious risks to individuals who consume them [26,163]. In high doses, narcotics can cause lethargy and coma with the possibility of causing death from respiratory depression. When physical dependence develops, withdrawal symptoms include an overwhelming desire to take the drug, anxiety, insomnia, muscle aches and, in extreme cases, cardiovascular collapse [164]. 

Moreover, musculoskeletal pain is the most frequent in the traumatic context and pain management is a crucial issue for athletes who train and compete at the highest performance levels [165,166,167,168]. In this respect, opioids are the drugs of choice for the treatment of pain states; however, depending on the pain state, therapy may include non-steroidal anti-inflammatory drugs (NSAIDs), anticonvulsants or antidepressants [169,170,171]. 

In sport, when involving pain, these substances are in fact capable of raising the pain threshold to such an extent that one can go so far as not even to notice any physical damage suffered or to perceive dangerous situations as harmless [172,173,174]. Necessarily treating an athlete’s pain while limiting the risk of adverse events is a challenging task. 

Opioids can be effective for pain management after a serious injury or orthopedic surgery, particularly when they are taken short-term and as prescribed. However, opioids are also highly addictive and are not intended for long-term use.

The most commonly used narcotics are morphine, which is the prototype of opiates: methadone and heroin, a synthetic drug, whose pharmacological actions are qualitatively comparable to those of morphine. 

Using opioids in these ways can set the step for addiction. A study published in *Sports Health* found opioid use to be widespread among athletes. For professional athletes, opioid use ranged from 4.4% to 4.7%. Meanwhile, high school athletes had opioid use rates of 28–46% over their lifetime [174].

The consumption of alcohol, tobacco and other drugs such as anabolic steroids and opioids has become a concern in high-performance athletes [175,176] since professional athletes are more exposed to drugs than the general population. Abuse of opioids can lead to long-term side effects (details in Table 3), with effects on and including hypothalamus, neuroendocrine functions, miosis, convulsions, depression respiration, cough, nauseant and emetic effects, cardiovascular and gastrointestinal effects. The development of tolerance and physical dependence with repeated use is a characteristic feature of all the opioid drugs. This disturbance often is revealed when administration of an opioid is stopped abruptly, resulting in withdrawal.

It has been appreciated for some time now that humans react differently to opioids. In fact, there are interindividual variabilities in opioid response. Furthermore, in any individual patient, a particular opioid may provide better analgesia than other opioids. In this respect, pharmacogenomics (PGx) is the study and clinical application of the role of genetic variation on drug response. The Clinical Pharmacogenetics Implementation Consortium (CPIC), in recent times, published a clinical practice guideline on the use of pharmacogenetic information for opioid therapy for pain control. This guideline includes recommendations for how *CYP2D6* genetic test results can be used to optimize therapy for codeine, tramadol and hydrocodone [177,178]. CYP2D6 is a member of the cytochrome P450 (CYP450) superfamily of enzymes and is the major enzyme responsible for metabolizing codeine and tramadol to their active metabolites, morphine and *O*-desmethyltramadol (aka M1), respectively. However, the *CYP2D6* gene is highly polymorphic, with genetic variants that can lead to very low (poor), normal or accelerated (ultrarapid) metabolism of the codeine, tramadol and other drugs for recent review [179,180]. CYP2D6 plays a role in the metabolism of many opioids; thus, *CYP2D6* polymorphisms can affect clinical efficacy and safety with opioid use. In addition to *CYP2D6,* other genes have been studied for their association with opioid clinical effect or adverse events, such as *OPRM1* and *Catechol-O-methyltransferase* (*COMT)* [179,180]. With regard to *OPRM1,* encoding MOR, more than 100 variant alleles exist in humans. The most studied single nucleotide polymorphism in *OPRM1* gene is *rs1799971* (118A > G, N40D); prevalence of variants is 40–50% in Asians, 15–30% in Caucasians, 1–3% in African-Americans, and experimental evidence suggested a potential role with increased risk for drug addiction [reviewed by 179]. In fact, murine model of human *OPRM1* A118G (A112G in mouse) displayed a weaker synaptic modulation of GABA and glutamate release onto VTA DA neurons [114], thus posing for a neurobiological mechanism that may underlie risk of developing addiction in carriers of such allele. *COMT* is a key regulator of catecholamines (adrenaline, noradrenaline and dopamine) methylation that influence their concentration in the pain perception pathways. *Rs4680* (1947 G > A, Val158Met) and *rs4818* (C > G) are the main polymorphisms in *COMT* gene, with a prevalence of variants of 50% and 40% in Caucasians, 28% and 17% in African-Americans, 44% and 31% in Asians, respectively [179]. However, compared to *OPRM1* polymorphism, there is a paucity of evidence linking *COMT* to interindividual variation in opioid sensitivity (reviewed by [179]).

Therefore, scientific evidence suggests that individual variabilities can be attributed to single nucleotide polymorphisms in genes involved in opioid pharmacodynamics and pharmacokinetics. Knowledge of these genetic factors through pharmacogenetic testing can help clinicians to prescribe a more adequate opioid that can offer patients with maximal clinical benefit and minimal risk of adverse effects.

As research unknots the various genetics, biochemical and receptor interaction differences of opioids in humans, it is expected that certainly obtainable, cost-effective testing will become available to aid clinicians in choosing an optimal opioid analgesic for an individual patient [178,181]. PGx-guided strategy for prescribing opioids can improve response rate, reduce side effects and increase compliance to treatment plans for pain [182,183].

### 4.1. Upcoming Epigenetic Effectors in Nociception 

Studies on epigenetics and pain remain largely preclinical and investigate the theoretical ability of epigenetics to alter the nociceptive pathways both in the periphery and centrally [21]. Mechanisms, actors and targets have been recently reviewed [22] and recently lncRNAs and circRNAs have been included in the list of the upcoming epigenetic modulators in nociception [184,185]. 

The main limitations of morphine use in pain control are analgesic tolerance and dependence. The effects of repeated injection of morphine on the expression of *H19*, *BC1*, *MIAT1* and *MALAT1* lncRNA were investigated in rats in specific brain areas (i.e., prefrontal cortex, hypothalamus, hippocampus, midbrain and striatum) [184]. Alterations in the expression of these lncRNAs were reported in midbrain, striatum and hypothalamus, suggesting that lncRNAs may be involved in the molecular mechanisms controlling morphine tolerance and dependence. 

Lastly, recent findings revealed the involvement of circRNA to cytokine pathway in order to mediate morphine analgesic tolerance [185]. In particular, *CircNf1* resulted in being highly expressed in the dorsal horns of morphine-treated rats and, acting as a sponge for *miRNA665* and *miRNA330-3p*, enhances the expression of the CXC motif chemokine ligand 12 (CXCL12), a homeostatic chemokine essential not only in the modulation of morphine analgesic effects in chronic pain but also in neurodevelopmental process, maturation of the CNS, neuronal toxicity and transmission, neuroglial interactions and nerve regeneration [186,187].

### 4.2. Upcoming Epigenetic Markers in Doping 

The abuse of pharmacological active substance which improve athletic performance may alter the expression of specific genes involved in muscle and bone metabolism by epigenetic mechanisms, such as DNA methylation, histone tails modifications and ncRNA production [188]. The presence of ncRNAs in biological fluids such as blood or saliva, and their adapted production to lifestyle, dietary supplementation or drug abuse make this new class of RNAs a potential powerful tool for the detection of athletes health status and for doping investigation [189]. 

## 5. Conclusions

Opioids are prescribed in a variety of settings for treatment of both acute and chronic pain. However, studies demonstrating benefits of long-term use of opioids to manage chronic, non-cancer pain are lacking. Moreover, the evidence clearly demonstrates that long-term use of opioids is associated with an increased risk of OUD and overdose, as well as a series of other adverse outcomes (e.g., cardiovascular events, fractures) [9,54,162,169].

Drug addiction is the consequence of long-lasting adaptions in brain reward and non-reward neuronal networks; hence, understanding how opioids affect neuroplasticity in mesocorticolimbic dopaminergic circuitry may be enable better knowledge of the process and the development of effective therapeutic strategies. Recently, most studies have been focused on the epigenetics signature of tissues and cells, which consists in a large plethora of chemical modifications in the DNA or histone proteins and functionally adapts the chromatin status and, in turn, gene expression to lifestyle and environment. Following opioid exposure, the epigenetic signature changes to different extents in the brain and periphery. However, the primarily maintenance medications of opioid addiction, i.e., methadone, also changes the epigenetic signature in terms of the DNA methylation status [190]. Hence, targeting the numerous writers and erasers of the epigenetic signature may be a promising tool to treat and heal from addiction and avoid relapses, but further studies are required in the field to study possible adverse, opposite or additive effects of different illicit drugs and therapies. Recently, epigenetics enlarged toward RNA world, confirming the well-known role of miRNAs in the control of pathways related to brain health and disease, but also revealing new actors such as lncRNAs, circRNAs or exosomes in the process of opioid addiction. However, most studies have been carried out in animal models, using single opioid compounds at doses sometimes far from human-relevant ones. Hence, there is a need for more studies in the field to cover the knowledge gaps. 

It is known that, generally, an opioid such as tramadol morphine or codeine (analgesics medication) is used widely in sport to treat pain and inflammation associated with injury [191], but interindividual variabilities in opioid response due to genetic factors occur [177]. Hence, pharmacogenetic testing can help clinicians to prescribe a more adequate opioid therapy to offer patients maximal clinical benefit and minimal risk of adverse effects and addiction. Opioid analgesics are commonly prescribed for acute and chronic pain but are subject to abuse. There is extensive literature on the role of medication in pain management. Opioids must be used under medical supervision, in order to relieve pain in serious illnesses such as cancer, to induce and maintain anesthesia or to contest an addiction (e.g., methadone program) [32].

A variety of effects can occur after a person takes opioids, ranging from pleasure to nausea and vomiting, severe allergic reactions (anaphylaxis) and overdose, in which the breathing and heartbeat slow or even stop. Medical doctors should be encouraged to keep their knowledge current about evidence-based practices for the use of opioid analgesics to manage pain, as well as specific steps to prevent and manage opioid overdose. However, the tailoring of medicine—on the road to personalized medicine—considers the molecular and genetic mapping of the individual, even if many factors still impede the smooth application of personalized medicine and represent challenges or limitations in its realization.

## Figures and Tables

**Table 1:** The “strong opioids” commonly used to treat moderate to severe pain.

Ligands	Opioid Receptors		
(Opioid Agonists)	MOR	DOR	KOR
Etorphine	+++	+++	+++
Fentanyl	+++		
Hydromorphone	+++		
Levorphanol	+++		
Methadone	+++		
Morphine	+++		
Sufentanil	+++		
DAMGO	+++		
Bremazocine	+		+++
Buprenorphine	P		
Butorphanol	P		+++
Nalbuphine.	- -		++
[D-Pen2,D-Pen5]-Enkephalin (DPDPE)		+++	
U50,488			++

P, partial agonist; +, major affinity; -, minor affinity.

**Table 2:** Very potent and highly selective opioid ligands for the treatment of opiate addiction.

Ligands	Opioid Receptors		
(Opioid Antagonists)	MOR	DOR	KOR
Naloxone	- - -	-	- -
Naltrexone	- - -	-	
CTOP D-Phe-Cys-Tyr-D-Trp-Orn-Thr-Pen-Thr-NH2	- - -		
Diprenorphine	- - -	- -	- - -
beta-funaltrexamine	- - -	-	++
Naloxonazine	- - -	-	-
nor-binaltorfimina	-	-	- - -
Naltrindole	-	- - -	-
naloxone benzoylhydrazone	- - -	-	-

+, major affinity; -, indicate minor affinity.

**Table 3:** Opioid receptors, site of action and their effects.

Receptor	Site of Action	Effects	References
MOR	Systemic	Analgesia, euphoria, constipation, respiratory depression, Nausea/vomiting	[32,38,39,40,41,42,43,44,45,46]
	Peripheral	Analgesia, constipation, reduced inflammation	[40,42,47,48,49,50]
DOR	Systemic	Analgesia, convulsions, anxiolysis	[51,52,53]
	Peripheral	Analgesia, Decreases colonic transit time (constipation),	[48,54,55]
KOR	Systemic	Analgesia, diuresis, dysphoria	[56,57]
	Peripheral	Analgesia, reduced inflammation, Visceral nociception antagonist	[25,39,58,59,60]

**Table 4:** Modulation of intrinsic excitability of dopamine neurons by opioid receptor activation.

Type of Receptor	Type of DA Neurons	Functional Response	Ref
MOR	VTA (tertiary cells)	Membrane hyperpolarization	[109,112]
	VTA (Ih+)	Increase firing (indirect effect)	[109]
	VTA (Ih+)	No effect	[109]
	VTA	Increase firing (indirect effect)	[104]
	VTA/SNpc	Inhibition of GABA-A IPSC	[113]
	VTA	Excited via CdCl2-sensitive cond.	[103]
	VTA--> NAcc med shell	Increased firing (indirect)	[114]
KOR	VTA--> BLA	Firing inhibition GIRK activation	[63,108,115]
	VTA principal cells	Firing inhibition GIRK activation	[109]
	VTA--> mPFC	Firing inhibition/GIRK activation	[108,110,111]
	VTA--> NAcc	No effect/Firing inhibition	
	SNpc	Mild Firing inhibition/GIRK activation	[111]
	VTA	Integration of excitatory inputs	[8]
DOR 1/DOR 2	VTA (DA and non-DA neurons)	K^+^-mediated hyperpolarization	[99]
	VTA	Ca^2+^-dependent excitation	[99]
	VTA	Inhibit GABA release	[115]
NOP	VTA	Big outward current	[116]
	VTA--> mPFC/NAcc	Small outward current	[117]
	VTA--> pACC	Small inward current	[117]

**Table 5:** Opioid addiction and emerging deregulated ncRNAs.

	ncRNA	Targets	References
Addictive-like behavior	*miRNA592-3p*	DRD2 REST	[148]
Heroin craving	*miRNA181a*	MeCP2	[147]
Heroin seeking	*miRNA218*	MeCP2	[145]
Morphine craving	*circTmeff-1*	*miRNA541-5p* *miRNA6934-3p*	[153]
	*miRNA592-3p*	TMFF1	[153]
Morphine-taking behaviour	*miRNA1298-5p*	-	[144]
	*miRNA32-5p*	*Dusp5* *Btg2* *Cldn11* *Dcaf6*	[144]
Morphine withdrawal symptoms	*miRNA132-3p*	-	[142]
Reconsolidation of cocaine-associated memory	*circTmeff-1*	*miRNA206* BDNF	[155]

BDNF: Brain-derived neurotrophic factor; *Btg2*: BTG anti-proliferation factor 2; *Cd69:* CD69 molecule; *Cldn11*: claudin 11; DRD2: dopamine D2 receptor; REST: RE1-silencing transcription factor; *Tmeff-1*: *tomoregulin-1*; -: not tested.

## Data Availability

Not applicable.
